# Synthesis of tolyl guanidine as copper corrosion inhibitor with a complementary study on electrochemical and in silico evaluation

**DOI:** 10.1038/s41598-022-18755-y

**Published:** 2022-09-01

**Authors:** Moaz M. Abdou, Mahmoud N. EL-Haddad

**Affiliations:** 1 Egyptian Petroleum Research Institute, Nasr City, Cairo, 11727 Egypt; 2 Chemistry Department, Faculty Science, Mansoura University, Mansoura, 35516 Egypt

**Keywords:** Electrochemistry, Organic chemistry, Chemical synthesis

## Abstract

A rapid and new synthetic route for *N,N*′-di-o-tolyl guanidine (**DTG**) synthesis from cheap materials is reported. The performance of **DTG** as an excellent inhibitor for delaying copper (Cu) corrosion with an efficiency higher than 98% at 20 × 10^−6^ M in an acidic solution was investigated via electrochemical measurements. These measurements included PDP, EFM, and EIS spectroscopy. The experimental data indicated that **DTG** has an efficient inhibiting effect on the corrosion of Cu in acidic media.The **DTG** was adsorbed on to the Cu surface via chemical adsorption and followed the Langmuir route. The PDP measurements revealed that **DTG** acted as a mixed inhibitor. Furthermore, EIS data showed that the **DTG** adsorbed through the metal/electrolyte interface. This resulted in forming a **DTG** protective layer on the Cu surface, thereby impeding the dissolution of Cu in the acidic solution. The corrosive solution containing the **DTG** inhibitor after immersion of the Cu specimen for 48 h, which promoted the formation of a complex between the Cu cation and **DTG**, was investigated via ultraviolet/visible spectroscopy. In addition, the formation of a **DTG** protective layer on the Cu surface was confirmed via scanning electron microscopy and atomic force microscopy analysis of the Cu surface morphology. Moreover, the active centers for interaction with the Cu surface in an acidic solution were investigated via in silico evaluation of **DTG**.

## Introduction

Copper (Cu) is considered a useful material due to its thermal stability, high electrical conductivity, and good mechanical properties. This material is used extensively for evaporators, conductors, condensers, pipelines, heat exchangers, and pipelines^1–4^. Although resistant to most environments, Cu is corroded in aggressive acidic media^5–7^, leading to economic losses^8–10^. Organic corrosion inhibitors with nitrogen hetero atoms exhibit good inhibition efficiency^11–13^. Their mode of operation involves the adsorption of ions or molecules on a metal surface (MS)^14–17^.

Current studies on Cu corrosion focus primarily on developing green corrosion inhibitors with good inhibitive performances without environmental pollution^18–20^. Guanidines are used in medicinal chemistry^21–25^ and are efficient corrosion inhibitors in different corrosive media^26–30^. These substances are highly functionalized compounds with three nitrogen atoms, two methyl donating groups, and π-electrons in the rings.

Based on the initial findings and our work on the synthesis and chemistry of nitrogen compounds^31–36^, we have developed a synthetic route for the synthesis of *N,N*′-di-o-tolyl guanidine (**DTG**). In the present work, **DTG**, which was selected as a corrosion inhibitor for Cu in an acidic solution, was studied via electrochemical, surface morphological, spectroscopic, and theoretical methods.

## Experimental procedure

### Materials and instrumentation

Analytical grade chemicals were used in this work. The solution of the **DTG** was prepared at different concentrations (5 × 10^−6^–20 × 10^−6^ M) in a solution of 0.5 M HCl. The working electrode was a cylindrical-shaped Cu rod (99.99% purity), welded with Cu-wire and embedded in resin with a 0.5 cm^2^ surface area (cover one side) open to exposure. Electrochemical study data were plotted and fitted in the Echem Analyst, version 5.50 (Gamry Instruments, Warminster, PA, USA). After immersion in test solutions, the surface morphology of the Cu samples was examined via SEM (JEOL JSM-53000) and AFM (N9498S Agilent Technologies) analysis.

### Synthesis and characterization of inhibitor DTG

To a magnetically stirred solution of *o*-toluidine **1** (1.07 g, 10 mmol) in EtOH (15 mL), a solution of cyanic bromide **2** (1.27 g, 12 mmol) in EtOH (5 mL) was added gradually at 0 °C until the exotherm subsided and was then refluxed for 1.5 h. The mixture was filtered and the filtrate was neutralized using 1 M NaOH. Subsequently, the precipitate was filtered off and recrystallized from aqueous EtOH, yielding **DTG** (2.16 g, 90%) as a white solid. R_f_ = 0.38 (hexane/EtOAc, v/v, 1/4) and M.p 175–177 °C. FT–IR (KBr) cm^−1^: 3452, 3386 (NH), 1631(C=C), 1575 (C=N).^1^H NMR (500 MHz, DMSO): *δ* 2.14(s, 6H), 5.08 (br s, 2H), 6.77–6.80 (t, *J* = 6.79 Hz, 3H), 7.01–7.04 (t, *J* = 7.02 Hz, 3H), 7.07–7.09 (d, *J* = 7.08 Hz, 3H). ^13^C NMR (125 MHz, DMSO): *δ* 147.69, 130.03, 126.16, 121.79, 121.24, 18.12. HRMS (ESI/QTOF) *m/z*: [M]^+^ Calcd for C_15_H_17_N_3_ 239.1422, found 239.1427.

### Electrochemical technique

Electrochemical measurements (PDP, EFM, and EIS spectroscopy) were performed using three-electrode cell systems with the Cu sample as the working electrode (WE; surface area (cover one side): 0.5 cm^2^). Platinum and saturated calomel electrode (SCE) served as the counter and reference electrodes. Before each measurement, the surface of the WE exposed to air was abraded with various grades (800, 1200, 1500, and 2000) of emery papers. Afterward, the electrode was rinsed with distilled water, degreased with acetone, rinsed with distilled water again, and dried with soft paper. The electrode was then dipped into the test solution for 45 min until a steady-state open circuit potential (E_ocp_) was achieved. All measurements were performed in a fresh test solution at a constant temperature of 30 °C ± 1 °C. PDP measurements were performed by polarizing the WE (scan rate: 0.5 mV s^−1^) at OCP in the range of ± 250 mV. EIS measurements were conducted at 100 kHz and 10 alternating current (AC) amplitude of ± 10 mV at the OCP. In addition, EFM measurements were conducted at two frequencies (2.0 and 5.0 Hz). The base frequency was 0.1 Hz and a potential disturbance signal of 10 mV.

### UV–Visible spectra measurement

UV–visible spectra measurements were performed on the **DTG** solution (20 × 10^−6^ M) before and after dipping Cu at 30 °C for 28 h. All spectra were recorded using a PG instruments T80 + spectrometer with a dual-beam operated at a band width of 1.0 nm in 190–1100 nm.

### Cu surface analysis (SEM and AFM)

In preparation for the morphology study, the abraded Cu samples were immersed in test solutions at 30 °C for 48 h. The samples were then removed, rinsed with deionized water, and dried. Properties of the **DTG** protective layer on the Cu surface were evaluated via SEM and AFM analysis.

### Quantum chemical calculations (***QCCs)***

Density functional theory (DFT) calculations were performed using the Gaussian program package (version 9.0; Pittsburgh, PA, USA). Furthermore, the **DTG** geometry optimization was performed using the B3LYP functional DFT level with the 6–31G^++^ (d, p) basis set in the aqueous phase.

## Results and discussion

### Synthesis of the DTG inhibitor

**Figure 1:**
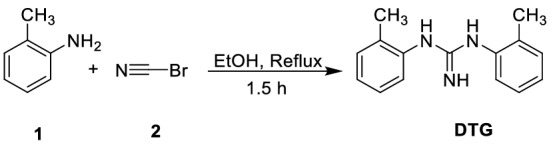
Synthesis of the **DTG**.

**Figure 2:**
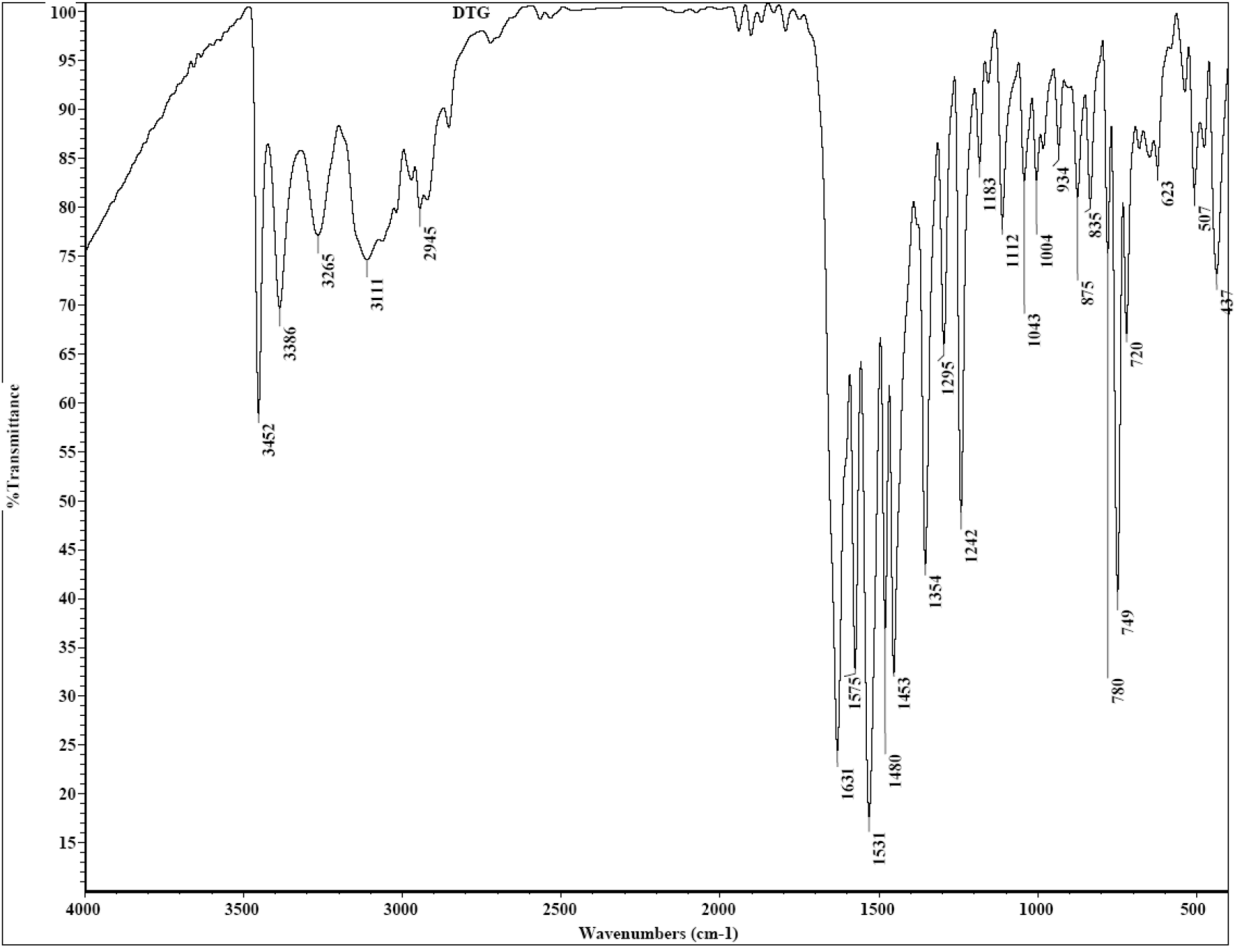
FT–IR spectrum of the **DTG** inhibitor.

**Figure 3:**
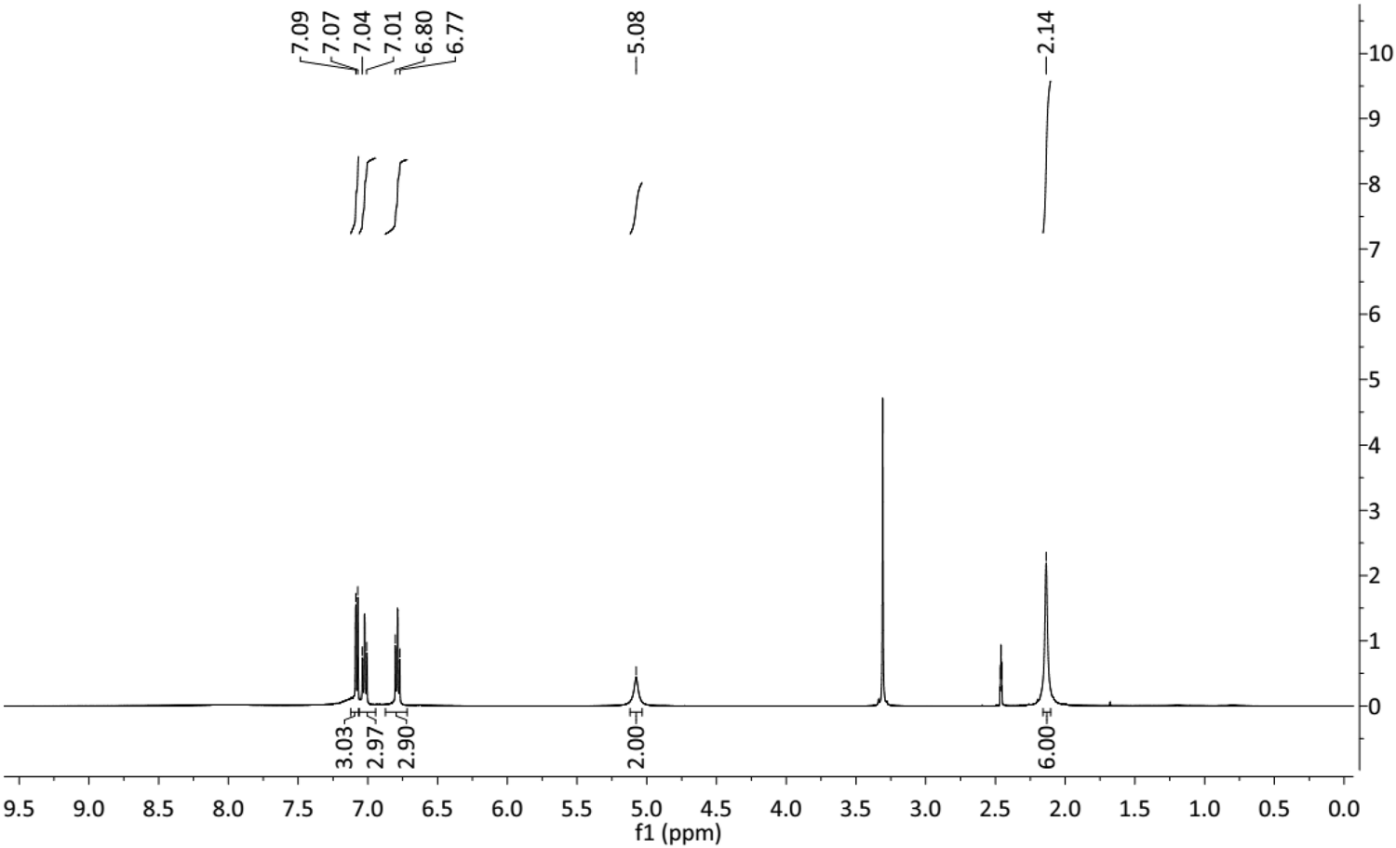
^1^HNMR spectrum of the **DTG** inhibitor in DMSO-*d*_*6*_.

**Figure 4:**
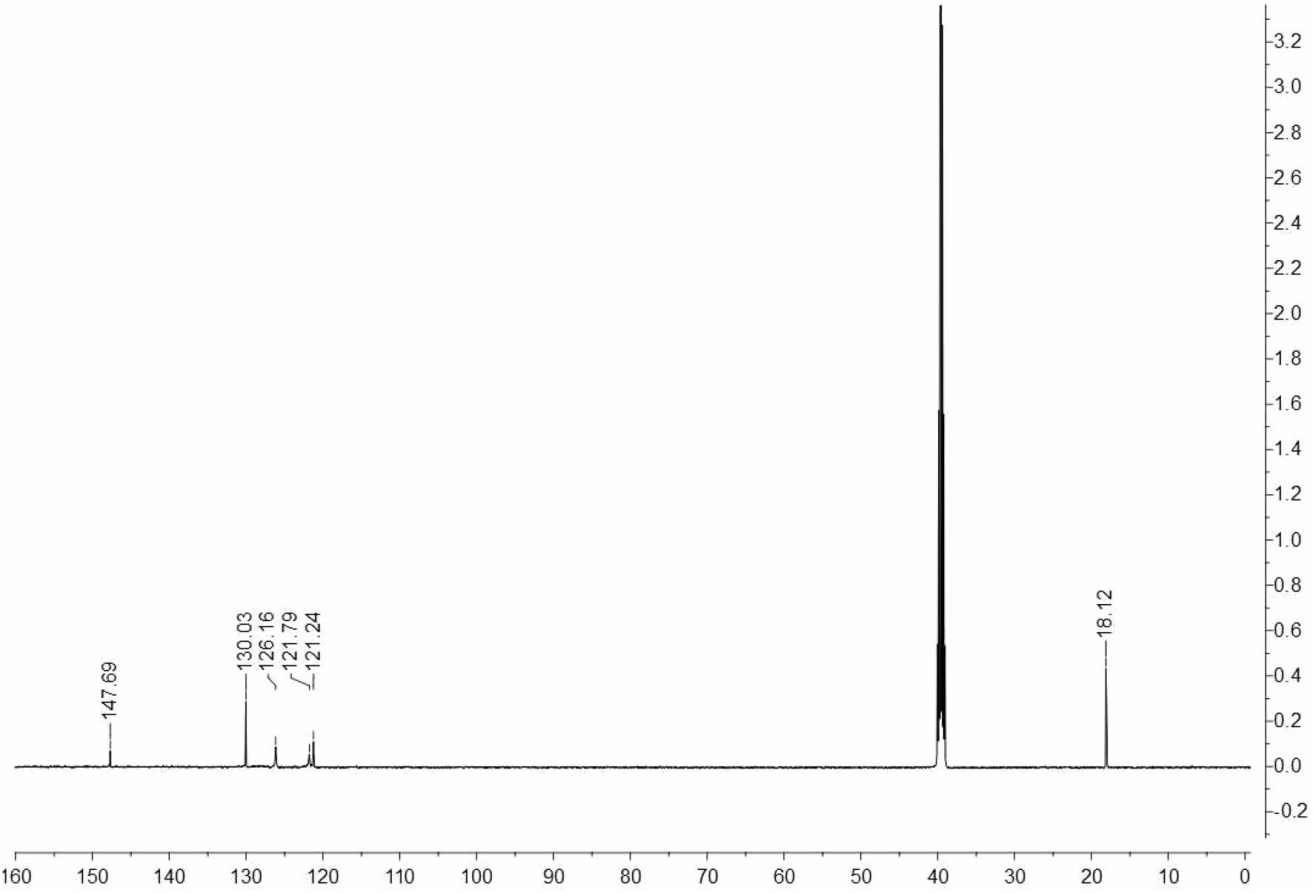
^13^C NMR spectrum of the **DTG** inhibitor in DMSO-*d*_*6*_.

The synthetic approach for **DTG** synthesis is depicted in Fig. 1. The reaction of *o*-toluidine **1** with cyanic bromide **2** in refluxing ethanol yielded the targeted **DTG** inhibitor (Fig. 1). The spectra of **DTG** concur with the proposed structure (*vide* Figs. 2, 3, 4). Various spectroscopic methods were used to characterize the structure of **DTG**. Characteristic bands occurring at 1631 and 1575 cm^−1^ in the infrared (IR) spectra are assigned to C=C and C=N bonds (Fig. 2). Similarly, the peaks at 3452 and 3386 cm^−1^ are attributed to the –NH group. ^1^HNMR spectra reveal two characteristic signals at 2.14 and 5.08 ppm, which are associated with Me and NH protons (Fig. 3). In the ^13^C NMR spectra, peaks associated with the C=N and C–NH resonate occur at 147.69 and 130.03 ppm (Fig. 4).

### Electrochemical measurements

#### PDP measurements

**Figure 5:**
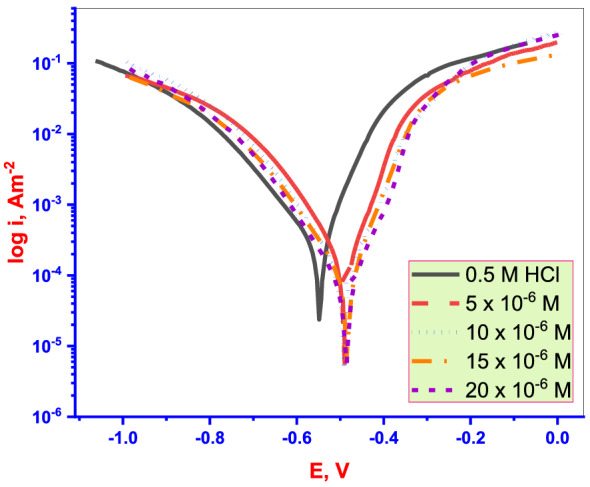
PDP curves for Cu in test solutions with/without of **DTG**.

PDP measurements depend on the **DTG** nature (anodic, catholic, or mixed-type inhibitor), mode of adsorption on the MS (physical, chemical, or mixture of both), and the mechanism of the **DTG** reaction with metal in the corrosive medium. Figure 5 shows the PDP curves obtained for Cu in test solutions with/ without different concentrations of **DTG** at 30 °C. The kinetic corrosion parameters such as the corrosion potential (E_corr_), I_corr_, the anodic Tafel slope (β_a_), and the cathodic Tafel slope (β_c_) are calculated from the analysis of the PDP plots (Table 1). The corrosion inhibition efficiency (*% IE*_*PDP*_) and the degree of surface coverage (θ) can be calculated from the I_corr_ values obtained via Tafel extrapolation of PDP curves and are given as follows^37^:

**Table 1:** PDP kinetic parameters for (**DTG**).

Conc. (µM)	I_corr_ (µA cm^−2^)	− E_corr_ (mVvs. SCE)	β_a_ (mV dec^−1^)	β_c_ (mV dec^−1^)	*θ*	%IE_PDP_
Blank	428	548	105	185	–	–
5 × 10^−6^	84.74	489	91.2	120.3	0.502	80.2
10 × 10^−6^	50.93	4491	92.2	131.2	0.881	88.1
15 × 10^−6^	25.68	485	74.8	128.5	0.938	93.8
20 × 10^−6^	8.56	484	88.5	119.8	0.983	98.3

1 $$ IE_{{PDP}}  = \left\lfloor {\frac{{I_{{corr}}  - I_{i} }}{{I_{{corr}} }}} \right\rfloor  \times 100 = \theta  \times 100. $$

I_corr_ and *I*_*i*_ are the corrosion current density of Cu electrodes in the test solutions.

Both anodic (Cu dissolution) and cathodic (H_2_ evolution reduction) have been inhibited after **DTG** was added to the corrosive medium (Fig. 5 and Table 1). This is suggests that **DTG** molecule acts as a mixed-type inhibitor. The *% IE*_*PDP*_ increases with increasing **DTG** concentration.

The E_corr_ values may also be used to classify inhibitors as cathodic, anodic, or mixed. When the difference in Ecorr values without and with inhibitors is greater than 85 mV 85 mV, the inhibitor molecules are classified as cathodic or anodic^38^. Table 1, demonstrates that the displacement of the E_corr_ values of the produced inhibitor molecules assembled on the surfaces of mild steel is less than 85 mV in the current study. As a result, the investigated **DTG** inhibitor acts as a mixed-type inhibitor.

However, the negative shifting of the E_corr_ values, and the β_c_ values are significantly higher than the β_a_ values in the presence of **DTG**. This suggests that the effect of **DTG** on cathodic H_2_ evolution is more prominent than that on anodic (Cu dissolution) in an acidic medium. In addition to, the *%IE*_*PDP*_ values increase, and the magnitude of I_corr_ decreases with increasing **DTG** doses (Table 1). This reduction indicates that the rate of electrochemical reaction decreased due to the formation of a protective **DTG** layer on the MS^39^.

#### EFM measurements

**Figure 6:**
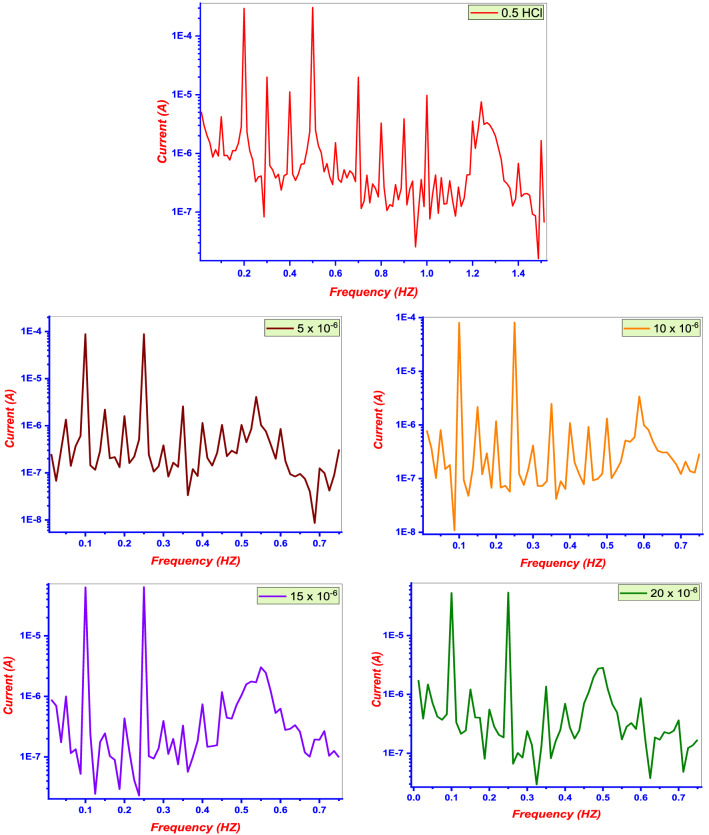
EFM spectra of Cu in test solutions with/ without of **DTG**.

The EFM technique is a rapid and non-destructive technique that determines the I_corr_ value without previous knowledge of the Tafel constants. The causality factors (CF-2) and (CF-3) indicate the validity of the EFM measurements. The EFM inter modulation spectra (current *vs.* frequency) of the Cu in 0.5 M HCl solution with or without various concentrations of the **DTG** are shown in Fig. 6. The EFM parameters (e.g., I_corr_, β_a_, β_c_, CF-2, and CF-3) were determined from the large spectra. The IE_EFM_ and *θ* values were calculated as follows:

2 $$ IE_{{EFM}}  = \left\lfloor {\frac{{I_{{corr}}  - I_{i} }}{{I_{{corr}} }}} \right\rfloor  \times 100 = \theta  \times 100. $$

I_corr_ and I_i_ represent the aforementioned corrosion current densities. The I_corr_ values decrease with increasing **DTG** concentration, owing to the protective thin film formed on the Cu surface. The CF-2 and CF-3 are close to their theoretical values (2 and 3, respectively), indicating the validity of the experimental EFM data (Table 2)^40^. Moreover, the EFM results are consistent with the PDP measurement results.

**Table 2:** EFM kinetic parameters of **DTG**.

Conc., (µM)	i_corr_, (µA cm^−2^)	β_c_, (mV dec^−1^)	β_a_, (mV dec^−1^)	CF-3	CF-2	*θ*	%IE_EFM_
Blank	436	143	91	2.85	1.90		
5 × 10^−6^	98.1	114	96	3.15	1.81	0.775	77.5
10 × 10^−6^	60.6	110	93	2.88	2.13	0.861	86.1
15 × 10^−6^	33.1	119	98	2.70	1.86	0.924	92.4
20 × 10^−6^	13.9	99	96	3.16	1.78	0.968	96.8

#### EIS measurements

**Figure 7:**
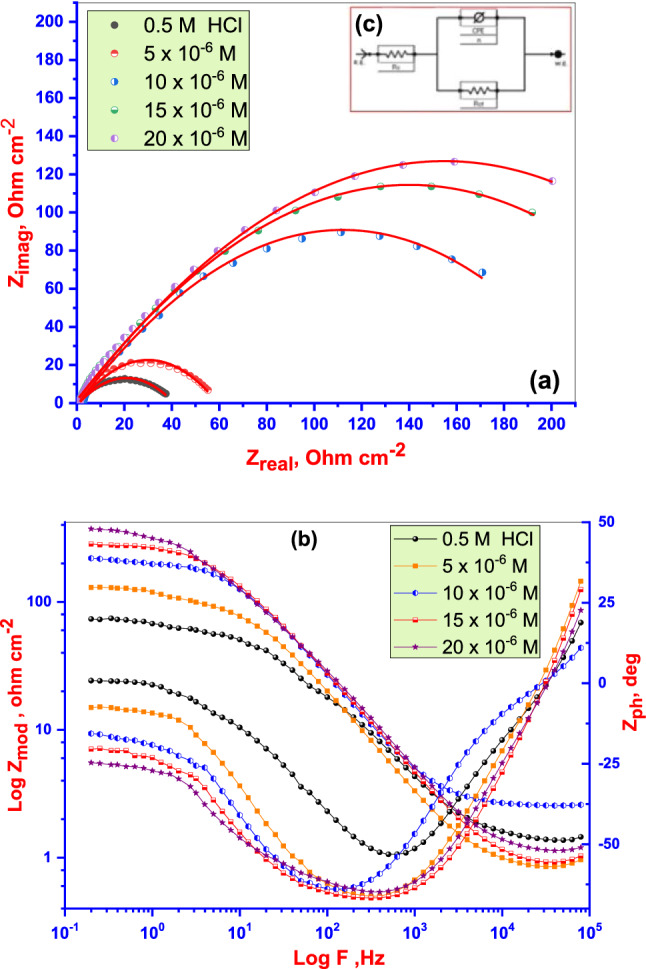
Nyquist plots (**a**), and Bode plots (**b**) for copper in 0.5 M HCl solution with/without of different concentrations of **DTG** with using a suitable equivalent circuit model (**c**).

EIS measurements were used to investigate the kinetic parameters for electron transfer reactions at the Cu electrode/electrolyte interface. Nyquist plots were obtained for the Cu electrode in 0.5 M HCl solution at an open-circuit potential after 45 min in immersion test solutions (Fig. 7a). A depressed semicircle corresponding to a single charge transfer process during the dissolution of Cu in the corrosive medium, which is unaffected by the existence of the **DTG**, occurs in the Nyquist plot.

Although a defined frequency was utilized to obtain the impedance at each data point, a Nyquist plot does not display any frequency value. To solve this deficiency, a Bode plot was created to show which frequency was utilized to generate a data point^41^. The Bode plots (Fig. 7b) reveal a single maximum at intermediate frequencies, with this maximum broadening in the presence of **DTG** due to the creation of a protective layer on Cu surface^42^. Higher values of phase angle and impedance for inhibited solution compared to uncontrolled solution reflect the inhibition effect of (EATPB). Furthermore, these values rise on the rise in the concentration of studied **DTG**. EIS plots were modeled and fitted using a suitable equivalent circuit (Fig. 7c).

The charge transfer resistance (R_ct_) and double-layer capacitance (C_dl_) values are deduced from the Nyquist plot analysis (Table 3). The (%IE_EIS_) and (*θ*) are calculated from the (R_ct_) (*vide* Eq. 3)^43^:

**Table 3:** EIS kinetic parameters for **DTG**.

Conc (µM)	R_s_ (Ω cm^2^)	n	R_ct_ (Ω cm^2^)	CPE_dl_ (μF cm^−2^)	*θ*	%IE_EIS_
Blank	2.09	0.578	410	243		
5 × 10^−6^	3.88	0.586	2015	45.0	0.815	81.5
10 × 10^−6^	4.11	0.598	2390	41.6	0.872	82.8
15 × 10^−6^	4.15	0.601	2950	33.7	0.932	86.1
20 × 10^−6^	4.18	0.621	8710	11.6	0.973	95.2

3 $$ IE_{{EIS}}  = \left\lfloor {\frac{{R_{{ct}}^{i}  - R_{{ct}}^{b} }}{{R_{{ct}}^{i} }}} \right\rfloor  \times 100 = \theta  \times 100. $$

R^i^_ct_ and R^b^_ct_ are the charge transfer resistance values with or without **DTG**, respectively.

The constant phase element (CPE) is applied to the state of capacitance (C) to represent a frequency-independent phase shift between an applied alternating potential and its current response. The CPE may be mathematically defined as follows^44^:

4 $${Z}_{CPE=\frac{1}{{{Y}_{0}\left(jW\right)}^{n}}.}$$

*Z*_*CPE*_, the impedance of CPE; *Y*_*0*_, a proportional factor; *W*, angular frequency; *j*, (− 1)^1/2^; and *n*, is related to (among others) the electrode surface roughness, distribution of reaction rates, and non-uniform current distribution^44^. Nyquist plots associated with a model containing (CPE) rather than (C) and the (R_ct_) correspond closely to the experimental results. The values of (*n*) corresponding to the 0.5 M HCl solution with **DTG** are lower than those of the solution without **DTG**, indicating the reduction of surface heterogeneity due to **DTG** adsorption onto the MS (Table 3)^44^. The R_ct_ values increase with increasing **DTG** concentration, while CPE_dl_ values decrease, leading to a maximum %IE _EIS_ (95.2%) at high concentration. This result indicates that **DTG** adsorption occurred at the metal/electrolyte interface, resulting in an adsorbed film on the Cu surface, which impedes the dissolution of the surface in the corrosive acidic medium^45^.

Thus, the results obtained from EIS corroborate the data obtained from PDP & EFM and indicate that adsorption films are formed. Owing to this adsorbed film, the number of active surface centers exposed to the corrosive acidic solution decreases and Cu dissolution and hydrogen evolution is delayed*.*

### Adsorption isotherm (AI) and thermodynamic parameters

AIs describe the interaction between both Cu surface and **DTG**. The efficiency of **DTG** depends mainly on their ability to adsorb onto the MS. The adsorption process includes the replacement of H_2_O molecules at a metal/electrolyte solution interface^46^ and is described as follows:

5 $$ {\text{Inh}}_{{({\text{sol}})}} + {\text{ nH}}_{{2}} {\text{O}}_{{({\text{ads}})}} \to {\text{Inh}}_{{({\text{ads}})}} + {\text{ nH}}_{{2}} {\text{O}}_{{({\text{sol}})}} . $$

**Inh**_**(sol)**_ and **Inh**_**(ads)**_ are the **DTG** in the solution and adsorbed on the MS, correspondingly, and n is the number of H_2_O molecules replaced by **DTG**. Various types of AI can be considered to elucidate the nature of the interaction between the **DTG** and the Cu surface^47^.

The experimental data obtained from PDP measurements are applied to fitting various AIsin our work. The Langmuir AI yields the best fitting of the PDP results. From this model of AI, the degree of surface coverage (*θ*) is related to the **DTG** concentration (C) as follows^40^:

6 $$ \frac{{C_{inh} }}{\theta } = \frac{1}{{K_{ads} }} + C_{inh} . $$

**Figure 8:**
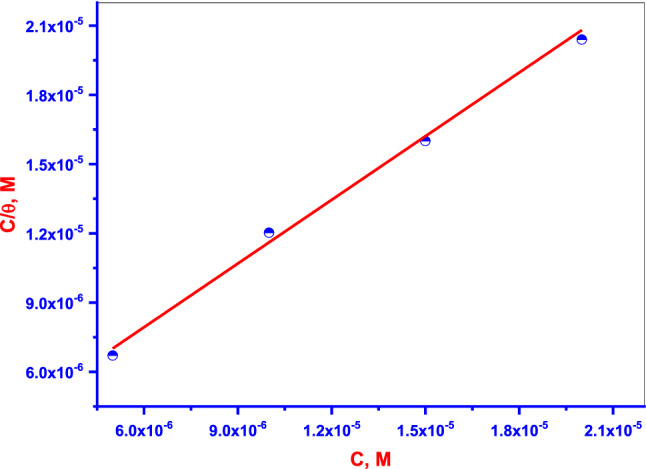
Langmuir AI for **DTG** at 30 °C.

Linear plots of C/*θ* versus C with strong correlation coefficients (R^2^) of 0.997, and a slope that is very close to unity for the **DTG** (Fig. 8) are obtained. The high value of K_ads_ (93 × 10^4^) from the plot's intercept indicates strong adsorption of the **DTG** onto the Cu surface. The equilibrium constant (K_ads_) of the adsorption process is associated with the standard free energy of adsorption (ΔG_ads_), which is given as follows^48^:

7 $$ K_{ads} = \frac{1}{{C_{solvent} }}\exp \left( {\frac{{ - \Delta G_{ads} }}{RT}} \right). $$

ΔG_ads_ values less than or equal to − 20 kJ mol^−1^ (more positive) are consistent with electrostatic interaction between the charged **DTG** and charged Cu surface, which occurs through physisorption. While, ΔG_ads_ values of approximately − 40 kJ mol^−1^ (more negative) or higher are related to charge or sharing electron transfer from the **DTG** to the metal surface via coordinate bond formation, which occurs through chemisorption^48^. The calculated value of ΔG_ads_ in the present study is − 44.2 kJ mol^−1^. This indicates that **DTG** is adsorbed physically on Cu surface in an acidic solution.

### Analysis of test solution (UV–visible spectrometry)

**Figure 9:**
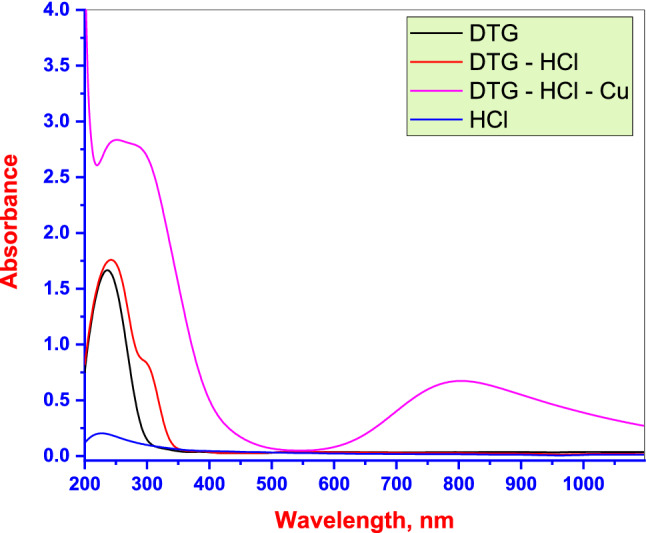
UV–visible spectra for the **DTG** (black color), 0.5 M HCl solution (blue color), 0.5 M HCl solution containing **DTG** (red color) before, and (violet color) after 48 h of copper immersion at 30 °C.

The possibility of complexation between the **DTG** and the Cu electrode in the corrosive test solution was investigated through UV–visible spectroscopic measurements. A **DTG** concentration of 20 × 10^−6^ M was employed (Fig. 9). Measurements were performed on Cu samples before and after 48 h of immersion at 30 °C in a 0.5 M HCl solution containing the **DTG**. An absorption band for the electrolyte (0.5 M HCl solution) is observed, while the band occurs at 233 nm in the absorption spectrum obtained for the **DTG** inhibitor may be associated with π–π* and n–π* transitions. An absorption band at 242 nm and a new band at 295 nm occur in the spectrum obtained for **DTG** in 0.5 M HCl solution. This suggests that the **DTG** structure is modified from uncharged to protonated form in the solution. However, a band at 801 nm is observed in the absorption spectrum of the solution resulting from Cu immersion in the 0.5 M HCl **DTG** containing solution. This result confirms that liqa complex is formed between **DTG** and Cu ions released during the corrosion reaction in the acidic solution^49^. Moreover, the ΔG_ads_ value is − 44.2 kJ mol^−1^. This confirms the chemical interaction of Cu ion dissolution with **DTG** in an acidic solution.

### Cu surface analysis (SEM and AFM)

**Figure 10:**
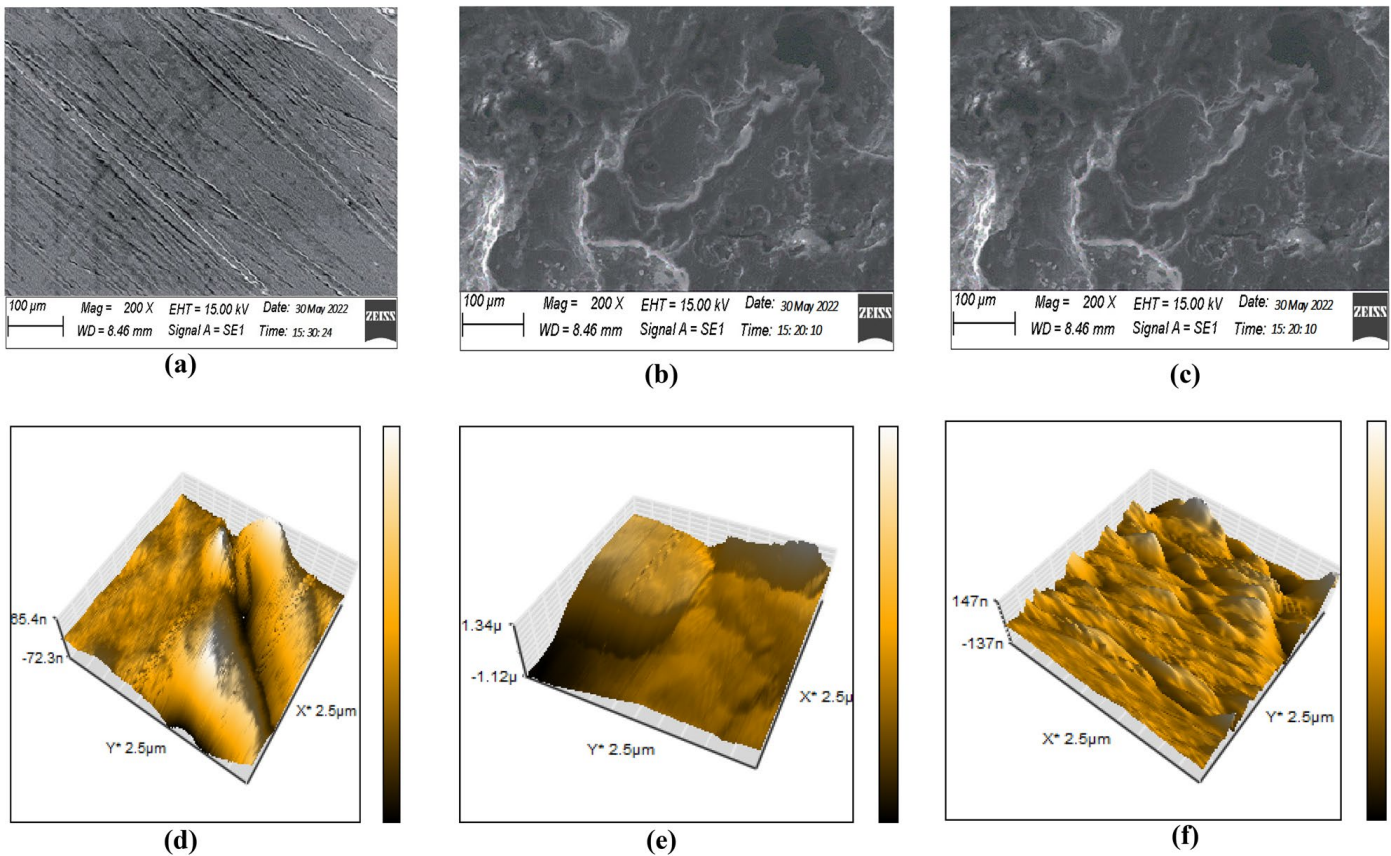
SEM (top row) and AFM images (bottom row) of the (**a**), (**d**) free Cu surface; surface in (**b**), (**e**) 0.5 M HCl and (**c**), (**f**) 0.5 M HCl with **DTG**.

Figure 10 shows SEM and AFM images obtained for abraded Cu samples and after immersion in test solutions for 48 h at 30 °C. The SEM shows that the Cu surface is smooth (Fig. 10a) before immersion in the test solution. In the absence of **DTG**, the surface is corroded in the solution (Fig. 10b) and becomes rough and porous. However, the addition of **DTG** (Fig. 10c) reduces the damage generated on the surface, confirming the inhibitory action^50^. The AFM images reveal an average roughness value (R_a_) of 32.379 nm (measurement surface: 2.5 × 2.5 μm^2^) for the free Cu sample (Fig. 10d). However, the R_a_ of the measurement surface increases to 80.231 nm after the sample is immersed in 0.5 M HCl solution (Fig. 10e). This is attributed to the attack on the MS by the acidic solution. In the presence of **DTG**, the R_a_ of the surface decreases to 41.780 nm (Fig. 10f). This indicates that the **DTG** could form a protective layer on the Cu surface, thereby delaying the attack of the corrosive medium on the MS^50^.

### QCCs

**Figure 11:**
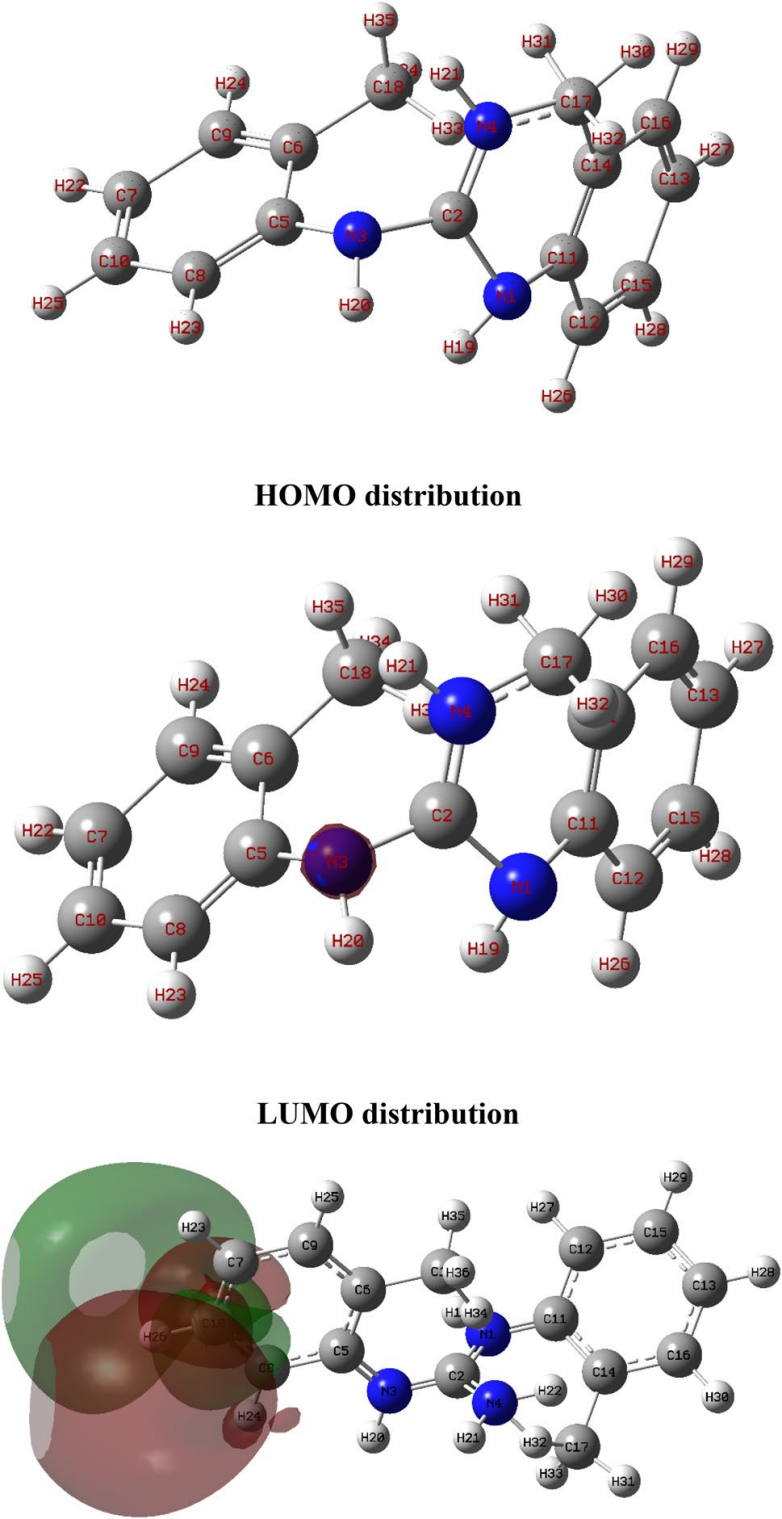
The optimized geometrical structure, HOMO and LUMO distributions of **DTG**.

To investigate the role of the DTG molecular structure and electronic properties in the interaction between DTG and MS, QCCs were performed using the DFT method. The presence of heteroatoms in **DTG** suggests a high tendency toward protonation in an acidic medium. Therefore, the influence of the molecular structure and electronic properties of the H^+^ form on the inhibition efficiency was investigated via the calculations. The optimized geometrical designs involving molecular orbitals (MO), highest occupied MO (HOMO), and lowest unoccupied MO (LUMO) energies are shown in Fig. 11 and the QCC results are shown in Table 4. Figure 11 shows that the HOMO location is distributed on the nitrogen atom (N3), and the LUMO location occurs on the benzene ring on the left side of the structure. This suggests that electrons are transferred and accepted from **DTG**. According to the frontier MO theory, the high energy value of E_HOMO_ (more negative) indicates the tendency of the molecule to donate e^−^ to acceptor molecules with an empty and low-energy orbital. Hence, the low energy value of E_LUMO_ (less negative) reveals the electron-acceptance tendency of the molecule (Table 4).

**Table 4:** QCC parameters for **DTG**.

E_HOMO_/eV	E_LUMO_/eV	∆E/eV	I_pot_./eV	E_aff._/eV	χ_inh_/eV	η_inh_/eV	ΔN
− 7.545	− 3.126	4.419	7.545	3.126	5.335	2.209	− 0.155

The energy gap (ΔE_LUMO−HOMO_) is an essential parameter associated with the **DTG** reactivity toward the MS. In the present study, the low value of (ΔE_LUMO−HOMO_) reflects the high inhibition efficiency of **DTG**, which improves molecule reactivity, thereby facilitating the adsorption of **DTG** on the MS^51^.The E_HOMO_ and E_LUMO_ of the **DTG** are associated with the ionization potential (I_pot_) and electron affinity (E_aff_), respectively^52^:

8 $$ I_{pot.} = \, - \, E_{HOMO} , $$

9 $$ E_{aff.} = - E_{LUMO} . $$

The absolute electronegativity (χ_inh_) and global hardness (η_inh_) of the **DTG** are obtained from *I*_*P*_ and *E*_*a*_ as follows:

10 $${\chi }_{inh}= \frac{{I}_{pot.}+{\mathrm{E}}_{aff.}}{2},$$

11 $${\eta }_{inh}= \frac{{I}_{pot.}-{\mathrm{E}}_{aff.}}{2}.$$

The interaction between **DTG** and MSs occurs when electrons flow from the **DTG** with lower χ_inh_ to the metal with higher χ_inh_ until the chemical potential becomes equal. The fraction of electrons transferred (ΔN) from the **DTG** to the MS is given as follows:

12 $$\Delta N=\frac{\left({\varnothing }_{Cu}-{\chi }_{inh}\right)}{2\left({\eta }_{Cu}+{\eta }_{inh}\right)},$$

where *ф* is the work function of the Cu surface (4.65 eV)^52^, while the absolute hardness of Cu (η_Cu_) was estimated as zero when *I*_*pot*._ = *E*_*aff.*_ for bulk metallic atoms.

The ΔN will occur from the **DTG** to the MS if ΔN > 0 and vice versa^52^. Table 4 shows that the value of ΔN is negative, indicating that **DTG** accepts the electrons from the Cu surface. This result supports the experimentally determined protection efficiency.

The Mulliken atomic charges of the **DTG** in the H^+^ form are listed in Table 5. As indicated in the table, the (1N, 3N, 4N, 5C, 8C, 9C, 10C, 12C, 14C, 15C, 16C, and 18C) heteroatoms have negative charges. This indicated that the **DTG** could be adsorbed onto the Cu surface by donating electrons from these atoms to the unoccupied d-orbitals of the Cu metal. The other atoms (2C, 6C, 7C, 11C, and 17C) have positive charges and can accept electrons from the 3d orbital of the Cu atoms. Therefore, an interaction occurs between **DTG** and MSs, thereby reducing the corrosion rate.

**Table 5:** Calculated Mulliken atomic charges and FIs of **DTG**.

Atom	Mulliken atomic charges	FIs	
		*ʄ* ^+^_*k*_	*ʄ *^*−*^_*k*_
1N	− 4.4546	0.098	0.021
2C	3.5658	0.094	0.087
3N	− 6.1454	0.097	0.011
4N	− 4.4125	0.097	0.044
5C	− 3.7244	0.074	0.016
6C	4.3232	0.098	0.084
7C	0.3075	0.096	0.084
8C	− 3.1541	0.076	0.025
9C	− 3.2323	0.074	0.021
10C	− 3.2116	0.074	0.023
11C	1.4456	0.010	0.086
12C	− 3.2215	0.072	0.035
13C	− 0.6341	0.011	0.009
14C	− 3.5974	0.072	0.012
15C	− 3.1424	0.074	0.011
16C	− 3.5472	0.070	0.036
17C	3.6822	0.094	0.011
18C	− 3.3589	0.071	0.036

The adsorption of the **DTG** on the MS occurs via the donor–acceptor (DA) interaction. The Fukui Index (FI) analyses help to identify the active sites of the molecule responsible for electrophilic (Eu) or nucleophilic (Nu) agents. The presence of the Nu or Eu center facilitates the **DTG**–MTS interaction. FIs for Nu and Eu attacks, which are determined through natural population analysis (NPA), are given as follows^53^:

13 $$ \int_{k}^{ + } { = q_{k} \left( {N + 1} \right) - q_{k} \left( N \right){\text{For}}\,{\text{Nu}}\,{\text{attack}}} , $$

14 $${\int}_{k}^{-}={q}_{k}\left(N\right)- {q}_{k}\left(N-1\right)\mathrm{For \; Eu \; attack},$$

where, *q*_*k*_(*N*), *q*_*k*_(*N − *1), and *q*_*k*_(*N* + 1) are the charge values of atom k in the neutral, cation, and anion states, respectively.

FIs for the charged species q_k_(N), q_k_(N − 1), and q_k_(N + 1) **DTG** are listed in Table 5. The high values of ʄ^+^_k_ and ʄ^−^_k_ indices are related to Nu and Eu attacks, respectively. As indicated in Table 5, NC and C atoms of the **DTG** are the most susceptible active sites for electron acceptance or donation. Most atoms are available, and susceptible centers for electron acceptance from the Cu surface (Nu attack) are associated with the high values of *ʄ* ^+^_*k*_ occurring for 2C, 6C, 7C, 11C, and 17C atoms. These atoms are available and favorable centers for electron acceptance from the MS (Nu attack). The high values of ʄ^−^_k_ are associated with the 1 N, 3 N, 4 N, 5C, 8C, 9C, 10C, 12C, 14C, 15C, 16C, and 18C atoms. These atoms are available centers for electron donation to the MS for chemical bond formation (Eu attack). Therefore, the **DTG** acts as an electron DA, thereby facilitating its adsorption on the Cu surface. The data obtained from QCCs matches the experimental data.

### Corrosion inhibition mechanism

The experimental and theoretical data showed that the adsorption mechanism is associated with the donor–accepter interaction between the **DTG** and Cu surface. In generally the adsorption of organic compounds may be classified into two types of interactions: physical adsorption and chemical adsorption. The physical adsorption involves the existence of both the electrically charged surface of the metal and charged species in the corrosive medium. The metal's surface charge is created by the electric field that exists at the metal/solution interface. In contrast, a chemisorption process includes charge sharing or charge transfer from the inhibitor molecules to the metal surface to establish a coordinate type of bond. This is feasible if the surface has both a positive and a negative charge. The presence of a transition metal with unoccupied, low-energy electron orbitals (Cu^+^ and Cu^2+^ in our study) and an **DTG** inhibitor with molecules with relatively loosely bound electrons or heteroatoms with a lone pair of electrons is required for the inhibition effect^54^. The **DTG** has N with a lone pair, and π-electrons enable chemical adsorption on the Cu atom with unoccupied sites. Π-antibonding orbitals of rings can accept the electrons from 4s or 3d of Cu atoms (retro-donation). On the other hand, Physical adsorption begins with Cl^−^ ions interaction, followed by electrostatic interaction between the positively charged N atoms in protonated form of **DTG** and the negative charge on the Cl^−^ ions adsorbed to the positively charged Cu surface in acid solution. This interaction leads to formation of a thin protective film that inhibits the Cu surface from interacting with corrosive species^55^.

## Conclusions

In this study, **DTG** is prepared and then characterized via different analytical and spectroscopic measurements.

The main results of the study are summarized as follows:

1. **DTG** exhibited good inhibition efficiency for Cu and was used as a mixed inhibitor based on potentiodynamic polarization results.
2. **DTG** adsorptive interaction with the Cu surface retards dissolution of the surface in the corrosive solution.
3. The DFT calculations of **DTG** indicate a good correlation between theoretical and experimental approaches.

## Data Availability

All data generated or analysed during this study are included in this published article.
